# Factors Contributing to the Health of 0- to 5-Year-Old Low-Birth-Weight Children in the United States: Application of the Multiple Disadvantage Model

**DOI:** 10.3390/ejihpe14010013

**Published:** 2024-01-09

**Authors:** Tyrone C. Cheng, Celia C. Lo

**Affiliations:** 1 School of Social Work, University of Alabama, Little Hall, Tuscaloosa, AL 35401, USA; 2 Peraton, Defense Personnel and Security Research Center, Seaside, CA 93955, USA; celiaclo@yahoo.com

**Keywords:** low birth weight, child health, chronic health problems, hospital use, poverty

## Abstract

This secondary data analysis of 1731 low-birth-weight children and their parents in the United States investigated children’s health and its associations with social disorganization, social structural factors, social relationships, health/mental health, and access to health insurance/services. The study drew on data from the 2021 National Survey of Children’s Health. Logistic regression yielded results showing low-birth-weight children’s excellent/very good/good health to be associated positively with parents’ education and health. In turn, child health was associated negatively with being Black, having a family income at or below the 100% federal poverty level, difficulty parenting the child, child chronic health condition(s), parent mental health, and substance use in the family. The implications of the present findings in terms of interventions promoting maternal and child health as well as participation in government assistance programs for low-income families are discussed.

## 1. Introduction

In 2011, over 24,000 infants died before their first birthday, and low birth weight (less than 2500 g) was the major cause of such infant mortality in the U.S. [1]. Prior studies showed that low birth weight was related to racism [2], being part of a racial/ethnic minority [2,3,4,5,6,7], poverty [2,3], food insecurity and a lack of health insurance coverage [7], having a single mother, the mother’s age and education [3,5], maternal smoking [4,5,7], parenting [8], stressful work [6], and being a female infant [3]. Furthermore, two prior studies reported that low birth weight was negatively associated with children’s growth in their early childhood [9,10]. Since these risk factors probably continue during infants’ growth, it is thus crucial to identify risk and protective factors in low-birth-weight children’s health in early childhood.

### Literature Review

**Figure 1:**
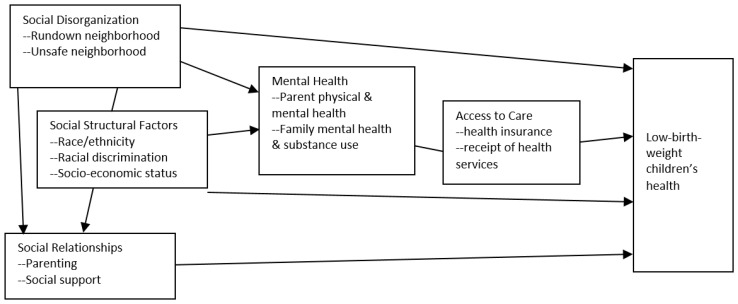
The multiple disadvantage model explaining low-birth-weight children’s health.

The present study applied the *multiple disadvantage model* to investigate factors in low-birth-weight children’s health in early childhood. The model proposes that the distress of socioeconomic disadvantages is likely to impair low-income parents’ mental and physical health, and further hinders their care of low-birth-weight children to such a degree that children’s health is adversely affected (see Figure 1). The model was applied to explain children’s health [11,12,13]. By applying the multiple disadvantage model, the present study investigated the impact of five socioeconomic disadvantages (social disorganization, social structural factors, social relationships, health/mental health, and access to care) on the health of low-birth-weight children in their early childhood.

Social disorganization, such as living in unkempt and unsafe neighborhoods, is a form of socioeconomic disadvantage. Some prior studies showed that living in impoverished neighborhoods and polluted environments increased the risk of having low-birth-weight infants [2,3]. Moreover, many prior studies on children in the general population have indicated that social disorganization is negatively associated with children’s health [12,13,14,15,16,17,18,19,20,21,22]. Hence, the researchers of the present study speculated that social disorganization factors like these would adversely affect low-birth-weight children’s health. 

Race/ethnicity and racial discrimination are two vital social structural factors related to low-birth-weight children’s health. The multiple disadvantage model acknowledges that historical and structural racism impact racial/ethnic minorities [23]. Racism-related frustration or distress experienced by parents in racial/ethnic minorities probably has an adverse effect on their parenting. Prior research reported that racism was related to infant mortality [24]; another prior study on low-income families showed that racial discrimination was negatively associated with children’s health [12]. In fact, within the first two years of life among low-birth-weight children, children from racial/ethnic minorities have a greater risk of death than White children have [25]. Other social structural factors—parents’ low education, under-employment, and low income—also heighten the risk of low-birth-weight infants’ mortality [24,26] and low-birth-weight children’s poor health within their first five years of life [27]. For low-income families who cannot afford sufficient food, their low-birth-weight infants’ mortality risk is escalated [28].

Supportive social relationships can lighten the distress of parents facing multiple socioeconomic disadvantages. With the support of strong social networks, parents generally demonstrate effective parenting [29] that promotes children’s health [20,30,31]. Prior studies of children in the general population demonstrated that poor health seemed to be associated with single-parent families [32,33]. Without social support, single-motherhood is likely to increase the risk of low-birth-weight infants’ mortality [26]. Furthermore, many low-birth-weight children have chronic conditions (e.g., asthma, poor cholesterol and blood pressure levels, and developmental delays) [27,34,35] that require constant parental attention and may generate parental distress. Also, low-birth-weight children tend to have negative affect and difficulties in social interactions [36,37]; consequently, many parents have difficulties in communicating with their low-birth-weight children and react with negative parenting behaviors [38]. In addition to low-birth-weight children’s chronic conditions, negative parent–child interactions are ultimately linked to these children’s poor health [39]. 

The multiple disadvantage model suggests that the challenges of socioeconomic disadvantages can affect parents’ physical health and mental health. A prior study indicated that parents’ physical health was associated negatively with the mortality risk of their low-birth-weight infant [24]. Moreover, parents with depression or anxiety may be unable to provide effective parenting and care their low-birth-weight children [40,41]. Under the stresses of multiple disadvantages and caring for low-birth-weight children, some mothers may use substances such as alcohol, tobacco, and/or drugs. In fact, mothers who smoke continue to negatively affect their low-birth-weight children’s health [42]. The present study assumed that the health of low-birth-weight children would have a positive relationship with their parents’ health and mental health, but a negative relationship with their parents’ substance use.

A lack of health insurance coverage is the fifth socioeconomic disadvantage affecting families with low-birth-weight children. Low-birth-weight children are more likely than other children to be covered by public health insurance [27]. Medicaid and Children’s Health Insurance Program (CHIP) are two public health insurance programs in the U.S. While Medicaid provides free or low-cost healthcare services to families whose incomes are at or below 133% of the federal poverty level [43,44], CHIP provides low-cost comprehensive healthcare coverage to uninsured children of families whose incomes are too high to be eligible for Medicaid but who cannot afford private health insurance [45,46]. In a study of the general population, publicly insured infants reportedly have a lower risk of mortality than other infants [24]. These findings imply that uninsured low-birth-weight children are likely to have poor health. Many low-birth-weight children with chronic health problems have great need of medical attention. In fact, prior studies showed that low-birth-weight children had a high tendency to use hospital care [47,48,49,50]. In other words, medical health insurance coverage for low-birth-weight children is crucial to their access to healthcare services and to their health.

The reviewed literature provided only a small number of U.S. studies of low-birth-weight children’s health in their early childhood. With the application of the multiple disadvantage model, the present study hypothesized that (1) low-birth-weight children’s health would be associated negatively with social disorganization (e.g., impoverished and unsafe neighborhoods), social structural factors (e.g., being a member of a racial/ethnic minority), social relationships (e.g., having a single mother, difficulties in parenting), parents’ mental health struggles and substance use, and children’s healthcare use; and (2) low-birth-weight children’s health would be associated positively with social structural factors (e.g., parents’ education, parents’ employment, and family income), social relationships (e.g., family support), parents’ health, and health insurance coverage.

## 2. Materials and Methods

### 2.1. Sample

This secondary data analysis of a nationally representative sample of 1,731 children was extracted from a public-use data set, the 2021 National Survey of Children’s Health (NSCH). Between June 2021 and January 2022, NSCH researchers interviewed 50,892 children and their caregivers in the U.S., gathering information on health status, insurance coverage, social relationships, family relationships, and neighborhood characteristics [51]. Of the 48,877 children (ages 0–17 years) with reports of their birth weight, 8.9% reportedly had low birthweight. The present sample was limited to children ages 0–5 years with low birth weight (less than 2500 g); their average birth weight was 2227.6 g. Furthermore, of all the children in the data set, no children reportedly had a birth weight below 1500 g. As a secondary data analysis, the present research received exempted approval from the institutional review board of the university.

### 2.2. Measures

The outcome variable *child health* was dichotomized as “excellent/very good/good” versus “fair/poor” (the reference). The original responses in the NSCH data set were: “excellent”, “very good”, “good”, “fair”, and “poor”.

The first group of two explanatory variables represented social disorganization factors. *Rundown neighborhood* (yes/no) was denoted if a parent’s neighborhood had “litter or garbage on street or sidewalk”; “poorly kept or rundown housing”; or “vandalism such as broken windows or graffiti”. *Safe neighborhood*, a continuous variable, measured how safe parents perceived their children to be in the neighborhoods they lived in. The range of responses were 4 (*definitely agree*), 3 (*somewhat agree*), 2 (*somewhat disagree*), and 1 (*definitely disagree*). 

The second group of explanatory variables represented social structural factors. *Racial discrimination* (yes/no) was denoted if a child had reportedly ever been treated or judged based on the child’s race/ethnicity. A child’s racial/ethnic background was indicated by four dummy variables—*Black*, *Hispanic*, *Asian*, and *other ethnicity/race*; *White* served as the reference group. *Parent education level*, a continuous variable, described the highest program completed by a parent, as follows: 1 (*8th grade or below*), 2 (*9th–12th grade*), 3 (*graduated high school or GED*), 4 (*vocational school*), 5 (*some college*), 6 (*associate degree*), 7 (*undergraduate degree*), 8 (*master’s degree*), 9 (*doctoral or professional degree*). *Employed parent* (yes/no) indicated if a parent had been an employee for 50 of the 52 weeks preceding the survey. Three dummy variables signified the ratio of each family’s income to the federal poverty level (FPL): *family income at or below 100% of FPL*, *family income at 101–200% of FPL*, and *family income above 200% of FPL* (the reference). The original NSCH data set provided the percentage of federal poverty level that a family’s income represented. 

The third group of explanatory variables represented social relationships and social support. *Single mother* (yes/no) indicated those who were not married or cohabiting with a partner, suggesting lack of access to the support of a spousal relationship. *Family cohesiveness* was a total score of two items: (a) whether their families drew on strengths family members possessed, and (b) whether their families talked with each other when facing problems. The response scale for the two items was as follows: 1 (*none of the time*), 2 (*some of the time*), 3 (*most of the time*), 4 (*all the time*). Higher total scores suggested greater family cohesiveness. The two items’ Cronbach’s alpha was 0.91. *Family support* (yes/no) denoted a parent’s emotional support received from a spouse/partner, other family members, and friends. *Difficulty of parenting the child* was the total score of three items regarding parents’ perception of caring for the child: “hard to care for”, “really bothers me”, and “angry with the child”. Responses included 1 (*never*), 2 (*rarely*), 3 (*sometimes*), 4 (*usually*), and 5 (*always*). The Cronbach’s alpha for the three items was 0.80. A higher total score suggested greater difficulty parenting the child. 

The fourth group of explanatory variables represented health and mental health factors. *Child chronic health condition(s)* (yes/no) indicated if a child had chronic difficulties/problems breathing, trouble with coordination or moving around, using her/his hands, physical pain, trouble swallowing, or difficult digestion during the 12 months preceding the NSCH interview. Two continuous variables, *parent health* and *parent mental health*, were measured with the same five responses provided by the NSCH: “excellent”, “very good”, “good”, “fair”, and “poor”. *Family mental health problem* (yes/no) denoted whether a child lived with a family member who was severely depressed, suicidal, or experienced other mental illness. *Family substance use* (yes/no) described if a child lived with a family member who abused alcohol and/or used other drugs. 

The fifth group of explanatory variables measured children’s access to healthcare. Three dummy variables were *private health insurance coverage*, denoting whether a child was covered by the parents’ employer-sponsored or privately-purchased health plan; *public health insurance coverage*, denoting if a child was covered by Medicaid or Medical Assistance; and *other health insurance coverage*, indicating if a child was covered by some other public health insurance program; *uninsured* served as the reference group. *Unavailability of health services* (yes/no) indicated that a child did not receive needed health services because they were not available in the area, their offices were closed, or they had no appointments available. *Hospital care* (yes/no) was a measure of a child’s hospital use during the 12 months preceding the NSCH interview. Finally, *female child* (versus *male child*), *child age* (in years), and *parent age* (in years) were demographic variables serving as controls in the modeling.

### 2.3. Data Analysis

The analysis excluded any children with birth weights at or above 2500 g. Excluding these children’s data introduced the possibility of selection bias during outcome modeling. A two-stage process addressed such a threat to our results’ validity. Firstly, we formulated a selection equation based on all employed explanatory variables plus an additional one, *food insecurity*, which indicated if a family could not afford sufficient food. This selection equation estimated, for each child included in our analysis, the probability of having low birth weight. That is, the selection equation obtained a *hazard rate* for each child. We incorporated *food insecurity* in our selection equation based on a prior finding that low-birth-weight infants were associated with food insecurity occurring in the year preceding data collection [7]. Moreover, including *food insecurity* furthered our pursuit of an identified outcome model [52]. Obtaining hazard rates provided an additional variable for the second stage of analysis, which constituted the final regression model. Each hazard rate stated the likelihood that the associated low-birth-weight child would be included in the final model [52]. 

Since the present study used a binary outcome variable, it employed STATA logistic regression featuring linearized variance estimations with robust standard errors. Moreover, the present study applied the sampling weights provided by NSCH researchers. A preliminary analysis indicated that *racial discrimination* generated a singularity because there were no cases in which a low-birth-weight child who experienced racial discrimination was reportedly in “fair/poor” health. Since the modeling was unable estimate the variable’s coefficient or odds ratio, our final analysis excluded the variable *racial discrimination*. On the other hand, preliminary assessment of multicollinearity problems indicated that the variables *private health insurance coverage* and *public health insurance coverage* generated low tolerance statistics (<0.4) and strong correlation (r = −0.72). We kept these two variables in final data analysis because understanding families’ and children’s medical insurance status was important to the present study. The final model yielded correlations among explanatory variables of −0.72 ≤ *r* ≤ 0.60.

## 3. Results

### 3.1. Descriptive Statistics

Descriptive statistics showed that a great majority (98.3%) of the low-birth-weight children had “excellent/very good/good” health (see Table 1). The average age of children and of parents was 3 years and 38.3 years, respectively; 53.7% of children were girls. Furthermore, 24.2% of these children lived in impoverished neighborhoods and the average score for safe neighborhoods was 3.6 (i.e., “somewhat agree”). Of the children, 58.6% were White, 10.7% were Black, 12.4% were Hispanic, 8.3% were Asian, and 10.0% were other ethnicity/race. In this study, parents’ average educational attainment was 6.1 (an associate’s degree), and 73.1% of parents were employed. Of the families in the sample, 15.4% had incomes at or below 100% of the FPL, 16.8% had incomes between 101% and 200% of the FPL, and 67.8% had incomes above 200% of the FPL.

Of parents in the present sample, 13.7% were single mothers. The average score for family cohesiveness was 10.6 (of 12.0 possible), and 79.7% reportedly received emotional support from family members and friends. On average, difficulty parenting the child was 5.1 (of 15.0 possible). Of the children, 24.4% had chronic health condition(s). Parents’ average health (3.8) and mental health (3.9) were good. Among children’s family members, 5.8% reportedly had mental health problems and 5.6% used substances.

Concerning insurance coverage, 64.9% of the insured children had private health insurance; 33.9% had public health insurance; and 3.4% had some other type of health insurance; only 3.5% of the children had no health insurance coverage. While only 1.8% of families reported the unavailability of health services, 6.6% of the children did use hospital care.

### 3.2. Multivariate Analysis Results

Multivariate analysis results confirmed that the hypothesized model differed significantly from the null model (Wald’s χ^2^ = 120.10, *p* < 0.01; see Table 2). The likelihood of children’s excellent/very good/good health was decreased among Black children (OR = 0.05; *p* < 0.05), but such a likelihood showed no association with other racial/ethnic minorities, living in impoverished neighborhoods, and residing in safe neighborhoods. While such a likelihood was positively associated with parent education (OR = 1.31, *p* < 0.05), such a likelihood had a negative association with family income at or below 100% of the FPL (OR = 0.26, *p* < 0.05). However, having a single mother, parent employment, and a family income between 101% and 200% of the FPL demonstrated no significant association with the outcome variable. 

On the other hand, children’s likelihood of excellent/very good/good health was diminished by difficulty parenting the child (OR = 0.66, *p* < 0.01) but had no significant association with family cohesiveness and family support. Although we observed a positive association (OR = 2.39, *p* < 0.01) between parent health and the likelihood of children’s excellent/very good/good health, child chronic health condition(s) (OR = 0.02, *p* < 0.01) had a negative association with such a likelihood. Moreover, reported parent mental health (OR = 0.31, *p* < 0.01) and family substance-use problems (OR = 0.26, *p* < 0.05) were found in this study to be associated negatively with a children’s likelihood of having excellent/very good/good health. Family mental health problems indicated no associations with the outcome variable. Finally, children’s likelihood of having excellent/very good/good health had a negative association with the use of hospital care (OR = 0.01, *p* < 0.01), but such a likelihood showed no significant association with health insurance status and the availability of health services. 

## 4. Discussion

Our study showed that over 98% of low-birth-weight children had “excellent/very good/good” health. A closer examination of the data revealed that 0.4% of low-birth-weight children had poor health and 1.3% of low-birth-weight children had fair health. Moreover, multivariate analysis findings showed that child health had associations in the positive direction with parents’ education and health and associations in the negative direction with being Black, having a family income at or below 100% of the FPL, difficulty parenting the child, child chronic health condition(s), parent mental health, and substance use in the family. No other variables showed significant associations with low-birth-weight children’s health.

Although prior studies in a single state [2,3] reported unkempt and unsafe neighborhoods’ positive associations with low-birth-weight outcomes, the present results indicated that living in such neighborhoods had no significant impact on low-birth-weight children’s health in their early childhood. However, close examination of the data revealed that the interaction term between living in a rundown neighborhood and child chronic health condition(s) (OR = 7.84 × 10^−7^, *p* < 0.01) yielded a strongly negative association with child health, implying residing in rundown neighborhoods worsens the health of low-birth-weight children with chronic health conditions in early childhood. Furthermore, these neighborhoods may still have long-term negative impacts on these children’s health and development in middle childhood and adolescence.

While this study found low-birth-weight children who were Black to be 95% less likely than low-birth-weight White children to have “excellent/very good/good” health, other low-birth-weight children from racial/ethnic minorities had no significant health difference with low-birth-weight White children. Moreover, consistent with the results of a prior study [27], the present study demonstrated that parents’ low education and having a family income at or below 100% of the FPL significantly reduced low-birth-weight children’s likelihood of “excellent/very good/good” health. Moreover, close examination of the data revealed that child chronic health condition(s)’ interaction terms with family income at or below 100% of the FPL (OR = 0.09, *p* < 0.05) and family income at 101–200% of the FPL (OR = 4.37 × 10^−7^, *p* < 0.01) yielded strongly negative associations with child health, implying low-birth-weight children with chronic conditions in low-income families would have an escalated risk of poor/fair health. On the other hand, although the present study’s finding on the insignificant impact of parent’s employment status was contrary to the result of a prior study [27], another close examination of the data showed that the interaction term between parent employment and family income at 101–200% of the FPL (OR = 17.15, *p* < 0.05) had a strong positive association with low-birth-weight children’s likelihood of “excellent/very good/good” health. In other words, parent’s full employment did help low-birth-weight children gain good health among many low-income families. One plausible explanation was that parents’ employment helped families obtain health insurance coverage. 

As expected, the present study found that low-birth-weight children who had chronic health condition(s), utilized hospital care, and were difficult to be taken care of were likely to have “fair/poor” health. Moreover, consistent with the finding of a prior study [42], the present results indicated that substance use in the family increased the likelihood of low-birth-weight children’s “fair/poor” health. These findings imply that many parents are the only caretakers of their low-birth-weight children with chronic health problems, and such responsibility may generate parental distress in caring for children. On the other hand, living with a spouse/partner, family cohesiveness, and family support had no significant association with children’s health. However, a close examination of the data indicated that the interaction term between single motherhood and family support had a positive association (OR = 8.66, *p* < 0.05) with the likelihood of “excellent/very good/good” health among low-birth-weight children. In other words, having a single mother with support from relatives and friends apparently improved low-birth-children’s health. 

Although parent health promoted low-birth-weight children’s health in the present study, its interaction terms with child chronic condition(s) and difficulty parenting the child yielded no significant association with child health. In other words, relying on parents’ good health alone probably is not sufficient to counter the challenges of taking care of these children. On the other hand, contrary to the findings of prior studies on small samples [40,41], the present research demonstrated that parent mental health had a negative association with low-birth-weight children’s health. One plausible explanation was that some parents had high anxiety because they vigorously maintained the good health of their low-birth-weight children. Although the present results found that both health insurance types and the unavailability of health services had no significant association with low-birth-weight children’s health, a closer examination of the data showed that the interaction term between private health insurance coverage and the unavailability of health services yielded a strong positive association (OR = 372.54, *p* < 0.01) with low-birth-weight children’s likelihood of “excellent/very good/good” health. Such a finding suggested that private health insurance probably covered regular care and gave more choices of providers and services despite the occasional unavailability of health services. 

One limitation in the present study was that the measures of rundown neighborhood and safe neighborhood for the families were not estimated with standardized scales. Generalizations of the results obtained from these proxy measures should be cautious. Another limitation was that the present findings were applicable to developed countries but not developing countries.

## 5. Conclusions

Applying the multiple disadvantage model provided a new perspective on understanding low-birth-weight children’s health and factors associated with their health. Most important is the implication from the present findings that interventions would most benefit low-income families with low-birth-weight children. 

To fortify the health of these children, community health workers and social workers should promote maternal health and help mothers gain access to prenatal care as well as continuous care through health coaching [53]; such interventions focus on risk factors such as maternal substance use, breastfeeding, and infant safe sleeping practices. Family-centered medical homes can be also helpful for these families (especially among Black families) by providing quality healthcare services and developmental screening [27,54]. 

To alleviate the distress of socioeconomic disadvantages such as poverty and social disorganization, government cash assistance, food stamps, and housing assistance can be helpful to low-income families with low-birth-weight children. To reduce mortality and improve the health of low-birth-weight children, the government should promote Kangaroo Mother Care (KMC) programs that require the mother to maintain continuous skin-to-skin contact with the infant as well as breastfeeding or breastmilk feeding at the facility or at home; moreover, home visits by health professionals and the support of other family members are crucial to children’s healthy development [55].

Future research studies might seek out the unique pattern of significant factors in the health of low-birth-weight children throughout their childhood. To understand low birth weights in terms of child development, future research might also involve longitudinal data accommodating an analysis of the full conceptual framework of the multiple disadvantage model. Use of the multiple disadvantage model would also allow future researchers to focus on the long-term impact of low birth weight on children’s specific medical and mental health conditions. Finally, future research in the same vein as the present study might investigate how Black low-birth-weight children’s health is affected by their parents’ participation in government assistance programs and medical home programs. 

## Figures and Tables

**Table 1:** Descriptive statistics of low-birth-weight children (*n* = 1731).

		Percent	Mean	Range	sd
Child health (excellent/very good/good)		98.3			
(fair/poor)		1.7			
Rundown neighborhood	(yes)	24.2			
	(no)	75.8			
Safe neighborhood			3.6	1–4	0.6
White		58.6			
Black		10.7			
Hispanic		12.4			
Asian		8.3			
Other ethnicity/race		10.0			
Parent education level			6.1	1–9	1.9
Employed parent	(yes)	73.1			
	(no)	26.9			
Family income at or below 100% of FPL		15.4			
Family income at 101–200% of FPL		16.8			
Family income above 200% of FPL		67.8			
Single mother	(yes)	13.7			
	(no)	86.3			
Family cohesiveness			10.6	3–12	1.8
Family support	(yes)	79.7			
	(no)	20.3			
Difficulty of parenting the child			5.1	3–15	2.0
Child chronic health condition(s)	(yes)	24.4			
	(no)	75.6			
Parent health			3.8	1–5	0.9
Parent mental health			3.9	1–5	0.9
Family mental health problem	(yes)	5.8			
	(no)	94.2			
Family substance use	(yes)	5.6			
	(no)	94.4			
Private health insurance coverage		64.9			
Public health insurance coverage		33.9			
Other health insurance coverage		3.4			
Uninsured		3.5			
Unavailability of health services	(yes)	1.8			
	(no)	98.2			
Hospital care	(yes)	6.6			
	(no)	93.4			
Female child		53.7			
Male child		46.3			
Child age (years)			3.0	0–5	1.5
Parent age (years)			38.3	19–75	9.3

Note: sd = standard deviation.

**Table 2:** Logistic regression results for low-birth-weight child health (excellent/very good/good) (*n* = 1731).

Variables	OR	RSE	90% CI
Hazard rate	6.83 × 10^15^ *	1.38 × 10^17^	22.67–2.06 × 10^30^
Rundown neighborhood (no)	1.92	0.90	0.89–4.14
Safe neighborhood	0.60	0.19	0.36–1.01
Black (White)	0.05 *	0.09	0.00–0.87
Hispanic (White)	0.71	0.68	0.14–3.46
Asian (White)	0.10	0.25	0.00–5.24
Other ethnicity/race (White)	0.90	0.69	0.26–3.20
Parent education level	1.31 *	0.18	1.04–1.65
Employed parent (no)	0.65	0.37	0.26–1.65
Family income at or below 100% of FPL (above 200% of FPL)	0.26 *	0.17	0.08–0.79
Family income at 101–200% of FPL (above 200% of FPL)	2.44	1.61	0.66–5.82
Single mother (no)	1.96	1.30	0.02–0.40
Family cohesiveness	1.17	0.14	0.96–1.43
Family support (no)	0.66	0.40	0.24–1.80
Difficulty of parenting the child	0.66 **	0.06	0.56–0.77
Child chronic health condition(s) (no)	0.02 **	0.02	0.00–0.12
Parent health	2.39 **	0.75	1.43–4.02
Parent mental health	0.31 **	0.11	0.17–0.55
Family mental health problem (no)	0.44	0.27	0.16–1.21
Family substance use (no)	0.26 *	0.17	0.09–0.76
Private health insurance coverage (uninsured)	0.46	0.48	0.08–2.55
Public health insurance coverage (uninsured)	0.09	0.13	0.01–1.08
Other health insurance coverage (uninsured)	4.60	6.66	0.42–49.87
Hospital care	0.01 **	0.01	0.00–0.09
Female child (male child)	0.81	0.44	0.33–1.99
Child age	0.92	0.21	0.63–1.33
Parent age	0.97	0.02	0.93–1.01
Wald’s χ^2^ =	120.10 **		

Notes: ** *p* < 0.01; * *p* < 0.05; OR = odds ratio; RSE = robust standard error; CI = Confidence Interval; reference groups are in parentheses.

## Data Availability

The data applied in this study are openly available on the U.S. Census Bureau website https://www.census.gov/nsch accessed on 11 July 2023.
